# 全肺切除术及支气管成形肺叶切除术在非小细胞肺癌治疗中的研究（附64例报告）

**DOI:** 10.3779/j.issn.1009-3419.2012.04.05

**Published:** 2012-04-20

**Authors:** 合利 杨, 少华 马, 潞艳 申, 克能 陈

**Affiliations:** 100142 北京，北京大学肿瘤医院暨北京市肿瘤防治研究所胸外一科，恶性肿瘤发病机制及转化研究教育部重点实验室 Key Laboratory of Carcinogenesis and Translational Research (Ministry of Education), Department Ⅰ of Thoracic Surgery, Peking University Cancer Hospital & Institute, Beijing 100142, China

**Keywords:** 肺肿瘤, 全肺切除术, 肺叶切除术, 生存率, Lung neoplasms, Pneumonectomy, Lobectomy, Survival rate

## Abstract

**背景与目的:**

全肺切除术是最早用于治疗肺癌的肺切除术，但自问世以来一直存在争议。本文旨在分析手术治疗的804例肺癌中的64例全肺切除术（pneumonectomy, P）或支气管成形肺叶切除术（bronchoplasty lobectomy, BPL）病例资料，以探讨其在肺癌治疗中的地位。

**方法:**

全组手术治疗肺癌共804例，重点分析其中64例P/BPL的临床特点，尤其对生存率进行分析。

**结果:**

64例肺癌中肿瘤侵犯肺动脉干（cT4）行全肺切除术25例（右侧6例，左侧19例）；因主支气管受累且距隆突 < 2 cm（cT3）行左全肺切除术4例，右肺上叶支气管成形肺叶切除术19例，左肺上叶及下叶袖状切除术各1例；因主支气管受累但距隆突≥2 cm（cT1/cT2）而行主支气管成形术13例；右全肺切除并半隆突成形1例（cT4）。64例P/BPL的患者1年、3年及5年生存率分别为93.6%、69.0%及45.1%，489例同期标准肺叶切除术生存率则分别为92.5%、77.3%及56.9%，两组5年生存率差异无统计学意义（*P*=0.226）。

**结论:**

标准肺叶切除术仍是非小细胞肺癌的主要术式，而P/BPL对部分高选择患者在全身治疗的支持下仍是可选术式。

## Abstract (en)

**Background and objective:**

Pneumonectomy was the initial successful surgery to treat lung cancer, but there has been being a lot of controversy since its inception. The aim of this study is to evaluate its potential role in the treatment of lung cancer through analyzing the survival for 64 cases underwent pneumonectomy or bronchoplasty lobectomy out of 804 cases underwent lung resection.

**Methods:**

Eight hundred and four cases of lung cancer underwent pulmonary surgery. We retrospectively reviewed the clinical data, especially foucused on the survival of 64 pneumonectomies or bronchoplasty lobectomies.

**Results:**

Of the 64 patients, 25 underwent pneumonectomy (6 right, 19 left) due to involvement of ipsilateral pulmonary artery trunk. Owning to involving ipsilateral main bronchus with the distance of tumor from carina < 2 cm, 4 pneumonectomies, 19 right upper bronchoplasty lobectomies, 1 left upper sleeve lobectomy and 1 left lower sleeve lobectomy were performed. Due to the ipsilateral main bronchus involvement with the distance of tumor from carina ≥2 cm, 13 cases underwent main bronchus bronchoplasty. One performed right sleeve pneumonectomy because of carina involvement. Overall 1-, 3- and 5-year survival rates of 64 pneumonectomies or bronchoplasty lobectomies were 93.6%, 69.0% and 45.1%, respectively, and that of 489 standard lobectomies done by the same surgery team in the same period were 92.5%, 77.3% and 56.9%, respectively. There was no significant difference in 5-year cumulative survival rates between the two groups (*P*=0.226).

**Conclusion:**

Although standard lobectomy remains the main type of surgery, pneumonectomy or bronchoplasty lobectomy is still one of option for the highly selective patients combining the support of induction chemotherapy.

外科治疗仍然是目前唯一有可能治愈较早期非小细胞肺癌(non-small cell lung cancer, NSCLC)的手段。外科手术目的在于切除受累肺组织及其区域引流淋巴结，从而控制局部病变以延长生存，并为精确分期进行全身治疗及其它补充治疗提供便利条件。外科治疗的原则为两个"最大"，即"最大"限度的切净肿瘤(R0切除)和"最大"限度的保存肺功能。自1933年Graham等^[1]^报道第1例肺癌肺切除手术(左全肺切除)之后，几经发展，目前认为解剖性肺叶切除术可以满足以上原则而优于全肺切除术，甚至近年来对早期肺癌肺切除术有逐渐缩小切除范围的趋势即采用肺叶以下切除^[2, 3]^。同时因全肺切除术尤其是右全肺切除术使肺功能损失巨大已有摒弃之势^[4]^。然而，临床上仍时有全肺切除术用于治疗肺癌。为此本研究对北大肿瘤医院单一手术组施行的804例肺癌手术进行回顾研究，重点就其中64例全肺切除术/支气管成形肺叶切除术的病例资料尤其是生存情况进行分析，以供参考。

## 1 资料与方法

### 1.1 病例资料

2000年1月-2011年12月北京大学肿瘤医院单一手术组治疗肺癌共计804例。全组患者术前均接受了胸部增强CT、纤维支气管镜、颈部超声、腹部超声及头颅MRI/CT检查。全肺切除术或支气管成形肺叶切除术的NSCLC共计64例，包括全肺切除术29例，支气管成形肺叶切除术34例，全肺切除并半隆突成形术1例。64例中男性56例，女性8例，中位年龄56岁，25例为直接手术，39例术前接受了诱导化疗。

对照组纳入标准：①既往无肿瘤病史；②解剖性肺切除术；③术后病理证实为NSCLC；④术后随访中未发生其它肿瘤。对照组共纳入行标准肺叶切除术的489例NSCLC患者，其中男性305例，女性184例，中位年龄62岁。

### 1.2 随访

术后2年内每3个月、5年内每6个月、5年后每年随访1次。随访方法主要以门诊复查为主，此次资料总结时则以电话、书信及家访相结合的方式加以补充。末次随访时间为2011年12月。

### 1.3 统计分析

采用SPSS 19.0统计软件进行数据分析。寿命表法计算累积生存率和中位生存时间，生存分析采用*Kaplan-Meier*法和*Log-rank*检验，以*P* < 0.05为有统计学差异。

## 2 结果

全组解剖性肺切除术553例，其中489例为标准肺叶切除术，64例为全肺切除术/支气管成形肺叶切除术，两组临床特征比较无统计学差异。64例NSCLC中因肿瘤侵犯肺动脉干(clinical T stage, cT4)行全肺切除术25例(右全肺切除术6例，左全肺切除术19例)；因主支气管受累且距隆突 < 2 cm(cT3)行左全肺切除术4例，右肺上叶支气管成形肺叶切除术19例，左肺上叶袖状切除术1例，左肺下叶袖状切除术1例；因主支气管受累，但距隆突≥2 cm(cT1/cT2)而行主支气管成形13例；右全肺切除并半隆突成形1例(累及隆突，cT4)。

全组死亡1例，为右全肺切除术后7天死于呼吸衰竭。64例肺癌患者临床分期由术前检查及术中所见决定，诱导化疗者手术切除范围按诱导治疗前临床分期决定。64例NSCLC临床分期为cⅠb期5例，cⅡa期5例，cⅡb期5例，cⅢa期45例，cⅢb期4例；术后病理分期(pathological stage)为p0期2例(yield pathological stage，yp0期2例)，pⅠa期1例(ypⅠa期1例)，pⅠb期5例(ypⅠb期1例)，pⅡa期2例(ypⅡa期1例)，pⅡb期11例(ypⅡb期9例)，pⅢa期38例(ypⅢa期21例)，pⅢb期5例(ypⅢb期4例)(表 1)。

**1:** 64例全肺切除术/支气管成形肺叶切除术的NSCLC的临床分期及病理分期 64 cases of pneumonectomy/bronchoplasty lobectomy in NSCLC- clinical and pathological stage

Items	Clinical stage								Pathological stage						
	0	Ⅰa	Ⅰb	Ⅱa	Ⅱb	Ⅲa	Ⅲb		0	Ⅰa	Ⅰb	Ⅱa	Ⅱb	Ⅲa	Ⅲb
Induction chemotherapy	0	0	0	0	4	32	3		2	1	1	1	9	21	4
Non-induction chemotherapy	0	0	5	5	1	13	1		0	0	4	1	2	17	1
Total	0	0	5	5	5	45	4		2	1	5	2	11	38	5
NSCLC: non-small cell lung cancer.															

489例标准肺叶切除术组和64例全肺切除术或支气管成形肺叶切除术组共计553例均无失访，随访率为100%。随访时间为2个月-140个月，平均随访时间为32个月。553例NSCLC的1年、3年、5年累计生存率分别为92.6%、76.0%及55.3%。全肺切除术/支气管成形肺叶切除术的64例NSCLC的1年、3年、5年累计生存率分别为93.6%、69.0%及45.1%，中位生存期为56个月。同期标准肺叶切除术的489例NSCLC的1年、3年、5年累计生存率分别为92.5%、77.3%及56.9%，中位生存期为61个月。标准肺叶切除术组与全肺切除术/支气管成形肺叶切除术组的5年生存率差异无统计学意义(*P*=0.226)(图 1)。

**1:**
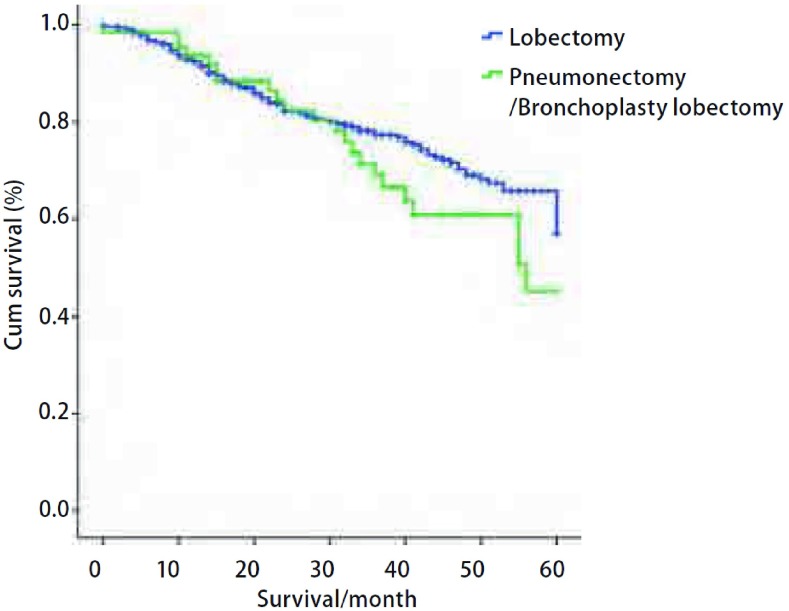
64例全肺切除/支气管成形肺叶切除与489例标准肺叶切除NSCLC生存曲线。全肺切除及支气管成形肺叶切除者的1年、3年及5年生存率分别为93.6%、69.0%及45.1%，标准肺叶切除者的1年、3年及5年生存率分别为92.5%、77.3%及56.9%，两组5年生存率相比无统计学差异（*P*=0.226）。 Survival curves of 64 pneumonectomies or bronchoplasty lobectomies and 489 standard lobectomies of NSCLC. The overall 1-, 3- and 5-year survival rates were 93.6%, 69.0% and 45.1% respectively in the 64 patients with pneumonectomy/bronchoplasty lobectomy, and were 92.5%, 77.3% and 56.9% respectively for 489 patients undervent standard lobectomy. There was no significant difference in 5-year cumulative survival rates between the two groups (*P*=0.226).

## 3 讨论

### 3.1 肺叶切除术仍然是肺癌的标准术式

肺切除术用于治疗肺癌始于1933年Graham^[1]^，该例全肺切除术乃是肺门的整块结扎切除。1934年Archibald^[5]^报道了解剖性全肺切除术，1937年Churchill^[6]^报道了肺叶切除术，1939年Churchill和Belsey^[7]^报道了肺段切除术。1950年Churchill等^[8]^研究比较了肺癌肺叶切除术与全肺切除术的治疗效果，结果显示肺叶切除术的5年生存率高于全肺切除术(19% *vs* 12%)，且肺叶切除术死亡率低于全肺切除术(14% *vs* 22.8%)，从而确立肺叶切除术为治疗肺癌的标准术式。此后肺叶切除术治疗NSCLC有大量研究报道。1995年Ginsberg等^[9]^前瞻性随机对照研究入组了276例患者，比较肺癌肺叶切除术与肺叶以下肺切除术的治疗效果，结果表明肺叶以下肺切除术并未降低手术并发症发生率及死亡率，并且术后局部复发率为肺叶切除术的3倍。此研究进一步巩固了肺叶切除术为肺癌标准术式的地位。然而全肺切除术就外科而言仍是安全的手术方式，临床时有应用。但自INT0139临床试验^[10, 11]^后，从肿瘤学的观点来看肺癌肺切除术的方式再次引起争议。该研究的对象为Ⅲa期患者，结果证明诱导化放疗后肺叶切除术较单纯化放疗可改善生存，而诱导化放疗后全肺切除术较单纯化放疗则不能改善生存。虽然本组64例全肺切除术或支气管成形肺叶切除术的生存率与肺叶切除术相似，但本研究的样本量较小，且该64例患者为高选择性病例。

### 3.2 右全肺切除术值得慎重

右全肺切除术的并发症发生率及死亡率明显高于左全肺切除术。一方面右全肺切除术后肺功能损失量明显高于左全肺切除术(41.5% *vs* 34%)，易发生呼吸衰竭^[12]^；另一方面右全肺切除术后血流动力学改变更为明显，易致肺动脉高压，进而出现心力衰竭^[13]^。Darling等^[14]^研究表明，右全肺切除术的死亡率高于左全肺(10.3% *vs* 3.3%)，右全肺切除术的并发症发生率亦高于左全肺(41.2% *vs* 38.9%)，如右全肺切除术后支气管胸膜瘘发生率明显高于左全肺(13.2% *vs* 5.0%)。Kim等^[15]^的*meta*分析研究了全肺切除术的围术期死亡率，共入选27个临床研究，其中15项研究显示右全肺切除术30 d病死率明显高于左全肺切除术(11% *vs* 5%)。临床上全肺切除术中行右全肺切除术者比例明显低于左全肺，Allen等^[16]^的研究中右全肺切除术仅占全肺切除术的38%，而左全肺切除术占62%。本组64例中右全肺切除仅7例(占24.1%)，且该7例中6例年龄均 < 55岁，仅1例为75岁男性，且于术后第7天死于呼吸衰竭，说明右全肺切除术死亡率较高，风险较大。

### 3.3 支气管成形肺叶切除术是替代全肺切除术的最佳术式

需要行支气管肺叶切除术者往往累及主支气管，就肿瘤根治性来讲应行全肺切除术。但是，支气管成形肺叶切除术因能更好的体现肺癌手术的两个"最大"原则而备受关注。理论上来讲，任何肺叶甚至肺段支气管均能行支气管成形叶/段切除术。但事实上，支气管成形手术在技术上容易度依次为右肺上叶、左肺上叶、左肺下叶、右肺中叶、右肺下叶。因而，文献^[17]^报道与临床应用最多者为右肺上叶，其次是左肺上叶，其它肺叶的支气管成形则因技术与解剖的原因往往招致术后的高并发症发生率而并不常用。Gómez-Caro等^[18]^报道55例袖状肺叶切除术中，右肺上叶袖状切除术28例(占51%)、左肺上叶袖状切除术16例(占29%)、左肺下叶袖状切除术5例(占9%)、右肺中叶袖状切除术4例(占7%)、右肺下叶袖状切除术2例(占3%)。本组右肺上叶支气管成形肺叶切除术19例，左肺上叶支气管成形肺叶切除术6例，右肺下叶支气管成形肺叶切除术3例，左肺下叶支气管成形肺叶切除术6例。支气管肺段成形肺切除术应该不宜用于肺癌的治疗，因为肺癌的标准术式为肺叶切除术而非肺段切除术，并且段支气管成形技术要求更高，在肺癌的治疗中有喧宾夺主之嫌。

### 3.4 全肺切除治疗肺癌的高选择性与多学科合作性

无论需全肺切除者还是支气管成形肺叶切除者，均说明为局部进展期病变。本组29例全肺切除术中均为T4或T3病变。文献^[19, 20]^报道NSCLC生存率随T、N分期升高而下降，即5年生存率T1a > T1b > T2a > T2b > T3 > T4(分别为77%、71%、58%、49%、31%、28%)，N0 > N1 > N2(分别为56%、38%、22%)。尤其是N分期，N2阳性者出现远处转移比率占40%，其中脑转移占34%，其3年生存率仅为26%^[21]^。因而本研究组的做法是对涉及全肺切除及支气管成形肺叶切除者先行诱导化疗^[22]^，本组术前化疗比率达60.9%(39/64)，但手术方式仍按化疗前的分期范围进行设计。除肿瘤学外全身情况是全肺切除术的另一考虑因素。一般来讲首选年龄 < 60岁，本组全肺切除术29例，中位年龄60.9岁，尤其是7例右全肺切除术中6例年龄均 < 55岁，仅1例年龄 > 60岁，且于术后7 d死于呼吸衰竭。

总之，肺切除术仍是目前公认的可治愈肺癌的方法，其中肺叶切除术因肺功能耐受好，远期生存好，被推崇为治疗肺癌的标准术式。而全肺切除术及其替代术式支气管成形肺叶切除术应在行全身诱导治疗的前提下对高选择人群慎重实施，或能取得令人鼓舞的效果。
